# The Application of Functional Imaging in the Diagnosis of Tumors

**DOI:** 10.1155/2017/3608912

**Published:** 2017-12-12

**Authors:** Xuelei Ma, Xiawei Wei, Shasha Li

**Affiliations:** ^1^State Key Laboratory of Biotherapy and Cancer Center, West China Hospital, Sichuan University and Collaborative Innovation Center for Biotherapy, Chengdu, China; ^2^Lab of Aging Research and Nanotoxicology, State Key Laboratory of Biotherapy and Cancer Center, West China Hospital, Sichuan University, Chengdu, China; ^3^MGH/HST Athinoula A. Martinos Center for Biomedical Imaging, Massachusetts General Hospital, Harvard Medical School, 149 13th Street, Charlestown, MA 02129, USA

In this special issue, the studies will give a more detailed description of functional imaging through several characteristic tumors. And the researches will find out more reliable evaluation background and more effectively potential targets through in-depth research on various functional imaging, such as PET/CT, PET/MRI, and ultrasound, which will contribute to the improvement of clinical value of functional imaging.

After long-term research, L. Domachevsky et al. further confirmed that the area under the portal vein was the most reliable site for evaluating the tumor background on PET/MRI. However, considering the range of FDG changes in the studies, the clinical difference in the area under the portal vein was expected to be confirmed.

In terms of nuclear medicine, various types of PET/CT and MRI are widely used in oncology. The review performed by C. L. Wright et al. showed that digital PET can improve the detectability of lesions in both tumor and nonneoplastic diseases and enhance the precision and accuracy of diagnosis and treatment compared with conventional PET.

Specifically, in N. Withofs et al.'s study, the overall detection rate of multiple myeloma by FPRGD_2_ PET/CT is lower than that of [^18^F]NaF/[^18^F]FDG PET/CT, but it might be useful in detecting bone marrow infiltration disease. Therefore, whether there is clinical and prognostic relevance of FPRGD_2_-positive patients remains to be further researched.

Somatostatin receptor-2- (SSTR2-) positive patients with neuroblastoma showed higher uptake of ^68^Ga-DOTA-TATE and were more sensitive to ^177^Lu-DOTA-TATE according to L. Zhang et al.'s research, which makes SSTR2 be a potential therapeutic target of neuroblastoma. D. Spiegelberg and J. Nilvebrant also discussed CD44v6, which is widely expressed in head and neck tumors and plays a similar role to that of SSTR2 in neuroblastoma.

Ultrasound is more widely used in clinical practice. The study by W. Ling et al. further confirmed the diagnostic possibility of rare Xp11 translocation renal cell carcinoma (RCC) by ultrasound. However, the bias due to small samples has to be considered, and we expect that the future studies will compensate for this shortcoming.

Functional imaging can be applied extensively in different types of tumors. Therefore, the continuous exploration and innovation are essential for enhancing clinical values in the future diagnosis and treatment, using either ultrasound or various imaging.

*Xuelei Ma* *Xiawei Wei* *Shasha Li*

